# Coverage Assessment and Target Tracking in 3D Domains

**DOI:** 10.3390/s111009904

**Published:** 2011-10-20

**Authors:** Noureddine Boudriga, Mohamed Hamdi, Sitharama Iyengar

**Affiliations:** 1 Communication Networks and Security Research Lab, University of Carthage, Ariana, 2083, Tunisia; E-Mail: hamdi.mm@gmail.com; 2 Robotics Research Laboratory, Louisiana State University, Baton-Rouge, LA 70802, USA; E-Mail: iyengar@csc.lsu.edu

**Keywords:** wireless sensor networks, coverage holes, 3D Voronoi diagrams, Vietoris-Rips complex

## Abstract

Recent advances in integrated electronic devices motivated the use of Wireless Sensor Networks (WSNs) in many applications including domain surveillance and mobile target tracking, where a number of sensors are scattered within a sensitive region to detect the presence of intruders and forward related events to some analysis center(s). Obviously, sensor deployment should guarantee an optimal event detection rate and should reduce coverage holes. Most of the coverage control approaches proposed in the literature deal with two-dimensional zones and do not develop strategies to handle coverage in three-dimensional domains, which is becoming a requirement for many applications including water monitoring, indoor surveillance, and projectile tracking. This paper proposes efficient techniques to detect coverage holes in a 3D domain using a finite set of sensors, repair the holes, and track hostile targets. To this end, we use the concepts of Voronoi tessellation, Vietoris complex, and retract by deformation. We show in particular that, through a set of iterative transformations of the Vietoris complex corresponding to the deployed sensors, the number of coverage holes can be computed with a low complexity. Mobility strategies are also proposed to repair holes by moving appropriately sensors towards the uncovered zones. The tracking objective is to set a non-uniform WSN coverage within the monitored domain to allow detecting the target(s) by the set of sensors. We show, in particular, how the proposed algorithms adapt to cope with obstacles. Simulation experiments are carried out to analyze the efficiency of the proposed models. To our knowledge, repairing and tracking is addressed for the first time in 3D spaces with different sensor coverage schemes.

## 1. Introduction

One among the main WSN issues that should be addressed while dealing with target tracking and monitoring applications, in 3D environments with obstacles, is area coverage. This is because a sensor can detect the occurrence of events or the presence of hostile targets only if they are within its sensing range. Coverage reflects how well a zone is monitored or a system is tracked by sensors. Therefore, the WSN detection performance depends on how well the wireless sensors observe the physical space under control.

Several metrics have been provided in the literature to measure the quality of coverage. Among these metrics, one can mention the following: (a) the number of coverage holes; (b) the proportion of uncovered area with respect to the area under monitoring; and (c) the so called Average Linear Uncovered Length (ALUL), which has been developed in 2D zones to estimate the average distance a mobile target can traverse before being detected by one sensor [1]. The ALUL can be used to assess the detection efficiency of the WSN in more general spaces. However, the major shortcoming of this approach is its heavy computational load making it non-conforming with the severe processing and energy limitations characterizing WSNs.

Obstacles in monitored 3D domains may complicate seriously the role of the monitoring sensors, increase their power consumption, and limit the coverage efficiency of the process providing coverage control [2,3]. Procedures set up to implement coverage control and target tracking efficiency should be optimal. They should take into consideration the geographic nature of the monitored area and cope with the number and the shape of obstacles.

This paper proposes a coverage assessment approach amenable to implement advanced target tracking functionalities. First, it provides a technique based on the concept of retraction by deformation applied to a special space, called the Rips complex, associated with the deployment of a set of sensors to develop a low complexity algorithm for locating coverage holes. Second, it constructs a collaborative mechanism to repair coverage holes, assuming that the sensors have mobility capabilities. Third, the paper builds on higher-order Voronoi diagrams to define an efficient scheme to coordinate tracking activities of single and multiple targets. To the best of our knowledge, this is the first time where retraction by deformation and higher-order Voronoi tessellations are used for hole assessment and target tracking in 3D domains with obstacles using sensors. The major contributions of this paper are as follows:

- The definition proposed to distributively reduce the Rips complex associated to the sensors is general, in the sense that it applies to a large variety of sensor, detection techniques, monitored domains, and obstacles.
- The proposed cooperative coverage repairing approach considerably reduces the uncovered areas and provides efficient handling of obstacles with respect to existing methods. The detection and localization of holes is done with low complexity.
- We show that the higher-order Voronoi tessellations we utilize are useful for performing multiple tasks including activity scheduling and coordination. In addition, we show that local coverage information, when gathered using the Voronoi diagram, can be used to implement coverage preserving mobility models.

The remaining part of this paper is organized as follows: Section 2 describes the state of the art of coverage control in various areas in general and in 3D spaces in particular. Section 3 surveys the definition of the mathematical objects needed for coverage and tracking control, the Vietoris complex and the Voronoi diagram and discusses the retraction by deformation. Section 4 discusses different schemes based on the Vietoris complex to detect and count the coverage holes in 3D domains, locate these holes, and repair them. It also defines a special procedure to reduce the complexity of the Vietoris complexes without modifying their topological properties. Section 5 sets up models for coverage assessment, sensor mobility, and target tracking. Section 6 analyzes the complexity of the algorithms constructed in this paper and sets some extensions of our results to more general types of sensors. Section 7 develops simulation experiments to evaluate the performance of a monitoring system implementing our techniques. Section 8 concludes this paper.

## 2. Related Work

Studies on coverage, holes, and boundary detection have been addressed using three main categories of techniques: geometric methods, statistical/probabilistic methods, and topological methods.

Studies using probabilistic approaches usually make assumptions on the probability distribution of the sensor deployment. Fekete *et al.* [4] assume uniformly randomly distributed sensors inside a geometric region for their boundary detection algorithm. Their approach hinges on the idea that the boundary nodes would have lower average degrees than that of the “interior” nodes and statistically provide a degree threshold to differentiate interior and boundary nodes. Kuo *et al.* [5] propose an error model for location estimation using probabilistic coverage, while Ren *et al.* [6] presents an analytical model based on probabilistic coverage to track moving objects in a densely covered sensor field. Most of probabilistic approaches have focused on the detection and tracking of objects in a sensor field. They did not address other related issues such as location of the holes, number of such holes and repairing.

A number of literature has addressed the static or “blanket” coverage. Dynamic or “sweeping” coverage [7] has been also a common and challenging task with applications ranging from security to housekeeping. Two primary approaches to static coverage problems in the literature. The first uses computational geometry tools applied to exact node coordinates. Such approaches are very rigid with regards to inputs: one, for example, must know exact node coordinates and must know the geometry of the domain to determine the Delaunay complex. To alleviate the former requirement, many authors have turned to probabilistic tools. For example, in [8], the author assumes a randomly and uniformly distributed collection of nodes in a domain with a fixed geometry and proves expected area coverage. Other approaches give probabilistic or percolation results about coverage for randomly distributed nodes. The drawback of these methods is the fact that uniform distribution of nodes may not be always realistic.

More recently, the robotics community has explored how networked sensors and robots can interact and augment each other: (see e.g., [9] for more details). There are several new approaches to networks without localization that come from research works in ad hoc wireless networks that are not unrelated to coverage questions. One example is the routing algorithm of [10], which generally works in practice but is a heuristic method involving heat-flow relaxation. This work investigates the issues of maintaining coverage and connectivity by keeping minimum number of sensor nodes to operate in the active mode. The authors show that if the radio range is at least twice the sensing range, then complete coverage implies connectivity. A decentralized and localized density control algorithm, called OGDC, is devised to control and maintain coverage and connectivity. However, their approach requires knowledge of node location. The authors claim that this requirement can be relaxed to that each node knows its relative location to its neighbors.On the other hand, Hsin and Liu [11] give methods for localizing an entire network if localization of a certain portion is known. They address the problem of target tracking in face of partial sensing coverage by considering the effect of different random and coordinated scheduling schemes. In their coordinated-coverage algorithm, a sensor might decide to sleep for some time after acknowledgments from its neighbor(s) that must be active. These decisions are not synchronized as individual sensors could negotiate with sponsors independently.

Since coverage verification is inherently a geometric problem, many research done in this area are based on computational geometry, and more precisely on the Voronoi Tessellation (and its dual, Delauney Triangulation). Motivated from the early success of the application of geometric techniques to cope with coverage problems (*Art Gallery Problem*), researchers have applied these techniques to ad-hoc distributed sensor networks ([12–15]).

The most important drawback of these approaches is that they are too computationally expensive to be implemented in real-time contexts. Another severe limitation is the impact of localization uncertainty on the performance of these approaches. These claims are well-documented in ([16]). In fact, to detect coverage holes, the locations of the sensors must be exactly known. Obviously, this cannot be always provided, especially when the sensing nodes are mobile. Moreover, equipping sensors with localization devices may considerably increase the deployment cost of the WSN and reduce its resources. In the following paragraphs, we summarize the methodologies, problems addressed and results of some of the recent, notable studies in the area of detection and coverage in wireless sensor networks.

Meguerdichian *et al.* [13] study the problem of computing a path along which a target is least or most likely to be detected. They provide an optimal polynomial time algorithm that uses graph theoretic and computational geometric (Voronoi diagram) methods. They address the issues of maximal breach path, maximal support path and provide best and worst case coverage using computational geometry. Delaunay triangulation was used to find the best-coverage path. In addition, deployment heuristics are provided to improve coverage. Since computational geometric methods require location information, the authors implement a location procedure prior to their coverage scheme. This procedure requires that a few of the deployed nodes (called beacons) must know their locations in advance (either from GPS or pre-deployment). Li *et al.* [14] uses local Delauney triangulation, relative neighborhood graph and the Gabriel graph to find the path with the best-case coverage.

Huang *et al.* [15] study the problem of *k*-coverage. They propose solutions to the *k*-UC and *k*-NC (Unit Disks and Non-Unit Disks) coverage problems which are modeled as decision problems whose goal is to determine if each location of a target sensing area is sufficiently covered. They present a polynomial-time algorithm with a geometric approach that runs in *O*(*nd* log *d*) time.

Ghrist *et al.* [17] use topological methods to detect insufficient sensor coverage and holes. In their seminal work on using homological concepts for addressing hole detection and coverage, their algorithm detects holes with no knowledge of their location. Although the approaches by Ghrist *et al.* have many desirable properties, the assumption of a static network and the centralized scheme are not suitable for dynamic networks.

## 3. Mathematics for Coverage and Tracking

The objective of this section is to provide a mathematical model for accurately gauging the coverage degree of a monitored domain in the 3D space **R**^3^ and repairing the coverage holes. This model uses the Vietoris Complex [6,8].

The following assumptions will be used in the next subsections: Let **M** be a bounded domain (or manifold) in **R**^3^ with non-empty boundary ∂*M*. The boundary is assumed to be an orientable topological surface (*i.e.*, a closed surface homeomorphic to some number of spheres and some number of connected sum of *g* tori, for *g* ≥ 1, [18]). Let *δ* : **R**^3^ × **R**^3^ → **R**^+^ denoting the Euclidean distance. We denote by *S* a set of sensors deployed in **R**^3^ to monitor *M*, and by |*S*| the number of these sensors. We will designate indifferently by *p* ∈ *S* the sensor in *S* and its location (*x_p_, y_p_, z_p_*) in **R**^3^. Let us notice, finally, that the sensors can be deployed inside **M** or outside it.

### 3.1. Voronoi Diagrams for Spherical-Detection Sensors

Let us assume that the sensors in *S* have identical covered area represented by a ball with radius *ρ*. For every pair *p, q* ∈ *S*, we denote by *B*(*p, q*) the plane, in **R**^3^ perpendicular to segment [*p, q*] and passing by its middle point and by *H*(*p, q*) the half space of **R**^3^ containing the *p* and delimited by *B*(*p, q*). Thus, *B*(*p, q*) and *H*(*p, q*) are expressed as follows:

(1) $$B\left(p,q\right)=\left\{x\in R^{3}/\delta \left(p,x\right)=\delta \left(q,x\right)\right\}.$$

(2) $$H\left(p,q\right)=\left\{x\in R^{3}/\delta \left(p,x\right)\leq \delta \left(q,x\right)\right\}.$$

We also denote by *H_M_* (*p, q*) and *B_M_* (*p, q*) the intersection of *H*(*p, q*) and *B*(*p, q*) with **M**, respectively.

The Voronoi cell generated by *p* ∈ *S* is nothing but the common area to the (*|S| −* 1) closed half spaces containing *p* involving the other sensors. Therefore, the Voronoi cell generated by *p* is expressed by:

(3) $$V_{S}\left(p\right)=\underset{q\in S\backslash p}{\cap }H\left(p,q\right)$$

The Voronoi cell of a sensor is convex and contractible. The common boundary of two Voronoi cells *V_S_*(*p*) ∩ *V_S_*(*q*) is included in *H*(*p, q*). It can be a plane, a half plane, an edge, a point, or an empty set. The Voronoi diagram associated to the set *S* of sensors deployed to monitor **M** is the unique subdivision defined in **R**^3^ by the Voronoi cells associated to all sensors. Thus, every cell of the subdivision contains the nearest neighbors defined in *S* for a sensor *p*. The Voronoi diagram of *S* is the set of point belonging to the all the Voronoi cell. Hence we have:

(4) $$V^{D}\left(S\right)=\underset{p\in S}{\cup }V_{S}\left(p\right).$$

In particular, the Voronoi diagram *V^D^*(*S*) has no vertices and no edges when the sensors are located at collinear points. In that case, the faces of the Voronoi diagram are parallel planes. In addition, one can notice that when *p* ∈ *S* lies on the boundary of the convex hull of *S*, then the Voronoi cell of *p* is unbounded in **R**^3^.

Since in this paper, we are rather interested in partitioning a domain **M** into cells according to *k*-nearest neighbors in *S*, for a given integer 1 ≤ *k* ≤ *n −* 1, we turn now to the definition of the High-order Voronoi diagrams, as they are useful concepts to define these sets and support target tracking. An order *k* Voronoi diagram is defined as follows: Let *T* ⊂ *S* containing *k* sensors, the *T*-generated cell is defined by

(5) $$V\left(T\right)=\left\{x\in R^{3}|\forall p\in T,\forall q\in S-T,\delta \left(x,p\right)\leq \delta \left(x,q\right)\right\}.$$

The order *k* Voronoi diagram is given by:

(6) $$V_{k}^{D}\left(S\right)=\underset{T\subset S,\left|T\right|=k}{\cup }V\left(T\right).$$

One can easily see that the order 1 Voronoi diagram $V_{1}^{D}\left(S\right)$ is just *V^D^S*, that *V*(*T*) can be empty, and that *V^D^*(*S*) induces a partition on the domain M into bounded components.

### 3.2. Vietoris-Rips Complexes

We consider a set of points *S* = {*v*_1_,..., *v_n_*} corresponding to the locations of a set of sensor nodes in a 3D space. For brevity, (*v_i_*)_1≤*i*≤*n*_ will be simply referring indifferently to as sensor nodes and points. We suppose that each sensor is capable of covering a disk of radius *r_c_* and communicate with the other sensors within a distance $r_{b}\leq \sqrt{3}r_{c}$. The total region covered by the sensor network can be represented by:

(7) $$\Gamma \left(S\right)=\underset{v_{i}\in S}{\cup }\Gamma_{v_{i},r_{c}}$$

where Γ_*v_i_,r_c_*_ = {*x* ∈ ℝ^3^ : ||*x − v_i_*|| ≤ *r_c_*}.

A *k*-simplex (or a simplex of dimension *k*) *σ* is an unordered set *σ* = {*v*_0_, *v*_1_, .., *v_k_*} ⊆ *S*, where *v_i_ ≠ v_j_* and *δ*(*v*ı*, v*j), for all *i ≠ j*. A face of the *k*-simplex *σ* is a (*k −* 1)-simplex formed by *k* elements (or vertices) of *σ*. Clearly, any *k*-simplex has exactly *k* + 1 faces. The collection of all k-simplices of S is called the abstract associated with Γ(*S*). In fact, an abstract simplicial complex *X* is a finite collection of simplices which is closed with respect to the inclusion of faces; meaning that, if *σ* ∈ *X*, then all faces of *σ* are also in *X*. It is noteworthy that a simplicial complex is a generalization of a graph; that is, the connectivity graph is nothing but the set of 1-simplices of the simplicial complex associated to a set *V* of points in the 3D space.

Now let us discuss the definition of the Vietoris-Rips complex. This complex captures the features related to connectivity and coverage of WSNs.

**Definition 3.1.** *(Vietoris-rips complex) Let S be a set of points in a 3D space and a given radius ε. The Vietoris-rips complex of S, denoted by R_ε_*(*S*)*, is the simplicial complex whose k-simplices correspond to unordered* (*k* + 1)*-tuples of points in S which are pairwise within Euclidean distance ε of each other*.

A subset of *k* + 1 points in *S* determine a *k*-simplex of for the Vietoris-rips complex if, and only if, each of these points lies within the intersection of the balls of radius *ε* centered at the other *k* points.

The reader, however, may wonder whether such topological structure can be computed in practice by tiny motes equipped with radio devices and limited storage capabilities. To answer this question, we propose a simple mechanism allowing a fully distributed construction of the Vietoris-rips complex. Through a 3-step broadcast of connectivity information, each sensor node can be aware of what simplices it belongs to, and what other simplices its neighbors belong to. To this end, we assume that every sensor node has a unique identifier (typically a layer-2 address) and has enough space to maintain a table of identifiers. The protocol performs as follows:

1. Initialization: Every sensor *v_i_* broadcasts its identity to its neighbors. Upon receipt of the message, each sensors builds the list, denoted by $\sum_{0}^{i}$, of 0-simplices formed by its neighbors.
2. Edge construction: Sensor *v_i_* appends its identity to the vertices in $\sum_{0}^{i}$ to construct the list, say $\sum_{1}^{i}$, of all 1-simplices it belongs to. It also determines the number *n_i_* of its neighbors. Then it informs its neighbors about the 1-simplices it built.
3. Simplicial iteration: On receiving the information from its neighbors, sensor *v_i_* starts building the lists $\sum_{j}^{i}$, 2 ≤ *j* ≤ *n_i_*, by simply adding appropriately the structures it has received to the ones it has already constructed.

An informal explanation of the construction algorithm is as follows. Simplices of higher dimension are constructed iteratively. In the first iteration, the 2-simplices are constructed by applying the following rule:

$$<v_{i},v_{j}>\in \sum_{1}^{i},<v_{i},v_{k}>\in \sum_{1}^{i},<v_{j},v_{k}>\in \sum_{1}^{j}\to <v_{i},v_{j},v_{k}>\in \sum_{2}^{i}$$

for every *i, j,* and *k*, provided that *i ≠ j, i ≠ k, j ≠ k*. The rules used for the following iterations are similar.

### 3.3. Homotopy and Retraction

Let *X* and *Y* be two topological spaces and *f, g : X → Y* be two maps (or continuous functions). We say that *f* and *g* are homotopic if there is a map *F* : [0, 1] × [0, 1] *→ X* such that

F(x,0)=f(x),F(x,1)=g(x),∀x,y∈X

Let *x*_0_ ∈ *X* be a given basepoint of *X*. A loop based on *x*_0_ is a map *α* : [0, 1] → *X*, such that *x*_0_ = *α*(0) = *α*(1). An equivalence relation on the set of all loops based at *x*_0_ can be defined by stating that loops *α*_1_ and *α*_2_ are equivalent if they are homotopic with respect to *x*_0_; meaning that there exists a homotopy *F* between *α*_1_ and *α*_2_ such that

$$F\left(0,t\right)=F\left(1,t\right)=x_{0},\forall t\in \left[0,1\right].$$

We denote the equivalence class of a loop *α* : [0, 1] *→ X* based at *x*_0_ by [*α*] and call it the based homotopy class of the loop *α*. The set of equivalence classes of loops based at *x*_0_ is denoted by *π*_1_(*X, x*_0_) and is called the fundamental group. It can be equipped with a multiplication defined by [*α*_1_] ★ [*α*_2_] = [*α*_1_.*α*_2_], for all loops [*α*_1_] and [*α*_2_] based at *x*_0_, where *α*_1_.*α*_2_ is the loop obtained by attaching *α*_1_ to *α*_2_. A second group of homotopy, denoted by *π*_2_(*X, x*_0_) can be defined as the set of homotopy equivalence classes of applications *β* : [0, 1]^2^ *→ X*, based at *x*_0_. It is an Abelian group, [19].

On the other hand, a map *f : X → Y* is called a homotopy equivalence if there is a map *g : Y → X* such that *f* ○ *g* is homotopic to the identity function in *X* and *g* ○ *f* is homotopy to the identity function *Y*. Thus, one can say that two spaces are homotopy equivalent if they have “the same shape”.

A deformation retraction of a space *X* onto a subspace *A* ⊆ *X* is a map *f : X* × [0, 1] → *X* such that:

f(x,0)=x,f(x,1)∈A,f(a,t)=a,∀x∈X,a∈A,0≤t≤1.

In other words, The subset *A* is a retraction by deformation of the space *X* if, starting from the original space *X* at time 0, we can continuously deform *X* until it becomes the subspace *A* at time 1 and deformation is performed without ever moving the subspace *A* in the process. It is obvious that, if *A* is a retraction by deformation of *X*, then *X* and *A* are homotopically equivalent.

Finally, let *K* be complex, a retraction filtration of *K* is a nested finite sequence of subcomplexes *K_i_*,

$$K_{0}\subseteq K_{1}\subseteq \ldots \subseteq K_{n}=K.$$

such that, for all *k* ≥ 0, *K_k_* is a retract by deformation of *K_k_*_+1_. Thus, it can be shown easily that, *K*_0_ and *K_n_* have the same type of homotopy and the same homotopy group.

Let *T* = {*p*_1_, .., *p_k_*} be a simplex and A *T*_1_ = {*p*_2_, .., *p_k_*} be one of its faces. Then *A* = *T* −(*T*_1_ − ∂*T*_1_) be the part of the boundary of *T* that is not internal to *T*_1_. Then *A* is a deformation retract of *T*.

Let *R*(*S*) be the Rips complex associated with *S*, repeating the process of retraction of simplexes that are on the boundary of *R*(*S*), with faces external to *R*(*S*), would lead to a filtration of *R*(*S*)*,* say *K_k_*, 0 ≤ *k* ≤ *n*, such that, for all *k* ≥ 0, *K_k_* is a retract by deformation of *K_k_*_+1_ and *K_k_*_+1_ is obtained from *K_k_* by adding one simplex, external to *K_k_* and belonging to *R*(*S*). The object *K*_0_ has no simplex with external face that is retractible.

## 4. Coverage Hole Management of Spherical Sensors

In this section, we propose a novel distributed technique to count the coverage holes of WSN using the retraction theory of spaces. In particular, we show that the Vietoris-rips complex associated with the WSN can be reduced to a simpler space that is tightly related to the number of holes.

In the following, let *D* ⊆ ℝ^3^ be a compact domain in the 3D space ℝ^3^ and ∂*D* be its boundary. We consider that *D* contains no obstacles. We also consider that a collection *S* = {*v*_1_, .., *v_n_*} is deployed over domain *D* and that the sensors are equipped with local communication and sensing capabilities. In fact, each sensor is capable of communicating directly with other sensors in its proximity (within a given distance *r_b_*) and has a limited sensing range *ε*.

### 4.1. Reducing the Vietoris-Rips Complex

We assume, in this subsection, a complete absence of localization capabilities and metric information, in the sense that the sensors in the network can determine neither distance nor direction. Under these assumptions, we are interested in designing distributed algorithms for coverage assessment and hole detection.

To this end, we need first to introduce a special procedure, called *Retract*, that reduces the size of the Vietoris-rips complex while keeping its type of homotopy. Repeating this procedure several times will eliminate all the 3-cells of the Vietoris-rips complex.

Let *R_ε_*(*S*) be the Vietoris-rips complex. Let {*v*_0_, ..., *v*_3_} be a 3-simplex in *R_ε_*(*S*) such that one of the 2-cell {*v*_0_, *v*_1_, *v*_2_} does not belong to another 3-simplex in *R_ε_*(*S*). If such a situation does not exist, then one can easily deduce that *R_ε_*(*S*) has no 3-cells. Let *X*_1_ and *A*_1_ be the set of points *x* ∈ *R_ε_*(*S*) belonging to simplex {*v*_0_*, v*_1_*, v*_2_} and the subset of *X*_1_ generated by the other two faces, respectively. Then its is easy to construct a map *h*_1_ : *X*_1_ *×* [0, 1] *→ X*_1_ such that:

$$h_{1}\left(x,0\right)=x,h_{1}\left(x,1\right)\in A_{1},h_{1}\left(a,t\right)=a,\forall x\in X,a\in A_{1},0\leq t\leq 1.$$

Map *h*_1_ can be easily extended to a map

$$\mathrm{Retract}:R_{\varepsilon }\left(S\right)\times \left[0,1\right]\to R_{\varepsilon }\left(S\right)$$

such that:

Retract(x,0)=x,Retract(x,1)∈A,Retract(a,t)=a,∀x∈X,a∈A,0≤t≤1.

where *A* is *R_ε_*(*S*) *− X*_1_) ∪ *A*_1_.

Repeating the map *Retract* several times will lead to eliminating all the 3-simplices in *R_ε_*(*S*). The map *Retract* can also be reapplied several times to delete all 2-simplices and 1-simplices that a free face. The resulting space, say $R_{\varepsilon }^{\mathrm{red}}\left(S\right)$.

**Proposition 4.1.** *Let S be a set of sensors. If R_ε_*(*S*) *is path-connected, then* $R_{\varepsilon }^{\mathrm{red}}\left(S\right)$ *satisfies the following properties:*

1. $R_{\varepsilon }^{\mathrm{red}}\left(S\right)$ *is homotopy equivalent to R_ε_*(*S*)
2. *the number of holes delimited by* $R_{\varepsilon }^{\mathrm{red}}\left(S\right)$ *is equal to the number of holes of the vietoris space R_ε_*(*S*)

*Proof.* Applying the map *Retract* several times helps creating a retraction filtration of *R_ε_*(*S*) such that:

$$R_{\varepsilon }^{\mathrm{red}}\left(S\right)=K_{0}\subseteq K_{1}\subseteq \ldots \subseteq K_{n}=R_{\varepsilon }\left(S\right).$$

where *n* is number of 3-simplices in *R_ε_*(*S*). Since, for every *i*, *K_i_* is homotopy equivalent to *K_i_*_+1_, we can deduce that $R_{\varepsilon }^{\mathrm{red}}\left(S\right)$ is homotopy equivalent to *R_ε_*(*S*).

The second statement of the theorem can be deduced from the following features:

- a holes is a path connected component that is surrounded by the delimiting space ( $R_{\varepsilon }^{\mathrm{red}}\left(S\right)$ and *R_ε_*(*S*)).
- Retracting a 3-simplex in *R_ε_*(*S*) may enlarge a hole but does not eliminate it.
- The retraction process does not create holes since it operates on the simplices that have free faces.

### 4.2. Counting and Locating Coverage Holes

To count and locate holes, we set up a 3-step algorithm. In the first step, we construct the external boundary of *R_ε_*(*S*). This is the subset of *S* containing all the nodes occurring on free faces and facing the boundary ∂*D* of the domain. In the second step, we define an algorithm that detects holes by progressively transforming the external boundary by retracting all its external simplices. In the third step, the following process is repeated: one external 2 simplex is deflated, the Retract map is applied several times to reduce appearing simplices with free faces, and the external boundary is updated. The number of iterations of this process gives the number of coverage holes.

#### 4.2.1. Constructing the Boundary of *R_ε_*(*S*)

Let us assume that the boundary ∂*D* of the domain **D** under monitoring can be seen (or detected) by the sensors in *S* and that the nodes in *S* broadcast periodically their unique ID numbers. The construction is based on the three following actions:

- Every sensor node detecting a boundary component of **D** or finding itself on an external facet sends this information to its neighbors.
- The information related to boundary detection, when received by sensors should be put together to form the external boundary of *R_ε_*(*S*), by simply allowing every sensor node to know which neighbor is on the external boundary.
- The nodes broadcast information related the external boundary of *R_ε_*(*S*) so that every node on the boundary can have a precise picture of the boundary.

#### 4.2.2. Counting Coverage Holes

Counting the coverage holes can be set up by an algorithm that repeats iteratively the following major procedures:

- Boundary retraction: Let *C_n_* be a n-simplex on the boundary of *R_ε_*(*S*) and *C*_*n*−1_ be one of its external faces, then *C_n_* can be retracted using the procedure Retract and the boundary is updated by adding a new node (the one in *C_n_* − *C*_*n*−1_), if *n* ≥ 2, or by deleting the node occurring in *C*_*n*−1_, if *n* = 1.
- Boundary deflation: When all the simplices on the boundary of have been retracted, a pre-selected node in *S* (in charge of the counter) selects one of the nodes of the new external boundary, withdraws it from the boundary, and increments the counter.

#### 4.2.3. Locating Coverage Holes

It is worth noticing that, when a deflation of a 2-simplex on the boundary *R_ε_*(*S*) is applied after retraction is complete, a hole is reduced from the coverage zone. This because the selected node, for deflation, is observing the hole, since it is one of the nearest nodes surrounding the reduced hole. Thus, this node can start the construction of the boundary of the reduced hole by determining the list of the nodes surrounding immediately the hole.

One can conclude, therefore, that any time a deflation is operated, a hole can be located by simply constructing its boundary using the nearest nodes to that hole.

### 4.3. Repairing Coverage Holes

Let us here assume that the 3D domain **D** under monitoring has no obstacles and let us denote by *χ* (*χ* = 4*πε*^3^/3) the volume of the area covered by a sensor and by *Vol*(*D*) the volume of **D**. One can state that the number *|S|* of sensors in *S* should be higher than the number *N*_0_ = *Vol*(*D*)/*χ* to be able to guarantee full coverage of **D**, at least after hole detection and coverage optimization. Therefore, we will assume in the sequel that this condition is satisfied. Finally, we assume that the sensors are able to move and detect the external boundary of **D**, when they are close to it, like in the above subsection.

Repairing holes aims at extending the coverage by eliminating the holes, or at least by shrinking considerably their size. An algorithm can be defined to this purpose. It can be built based on the following general rules:

- A node detecting the external boundary ∂*M* should keep seeing the boundary when it moves.
- A node on the external boundary of *R_ε_*(*S*) should move towards the uncovered area, when it does not see the boundary.
- When two neighbor nodes on the external boundary of *R_ε_*(*S*) are separated by a distance higher than a predefined threshold, say *θ*_1_, and one of them is not seeing the boundary of **D**, then the sensor unable to see the boundary asks its successor (*i.e.*, a neighbor involved in the retraction of the simplex containing this sensor) to move towards the external boundary.
- A node seeing the boundary should inform its neighbors so that they can move accordingly.
- When the distance between a sensor *s* and its neighbors on the boundary of a hole is lower than a predefined value, say *θ*_2_, then *s* should move in the opposite direction of the hole, while the other sensors should move towards the hole so that when they see each other, *s* can withdraw itself from the minimal surface after informing its neighbors.
- A node on the external boundary, finding itself unable to move informs, its successor to move towards its direction.

## 5. Target Tracking in 3D Domains

In this section, we use 3D Voronoi diagrams to optimize sensor coverage and target tracking performance. We first propose a strategy to measure the uncovered zones of the monitored region. Then, we develop two mobility models that provide target tracking using order k Voronoi diagrams and optimize the coverage ratio of a zone using Voronoi cells. Finally, we extend these models to multiple target tracking. We assume in this section that the sensors have spherical coverage. The vector-guided case can be addressed using similar techniques.

### 5.1. Measuring Uncovered Areas

Assume that a location *x* within the surveillance area is not covered by any sensor. Let ℒ(*x*, *θ*) define the Linear Uncovered Length (LUL) at location *x* with direction *θ*. This is the undetected path length of a target traveling from location *x* with direction *θ* = (*θ*_1_, *θ*_2_), for 0 ≤ *θ*_1_ ≤ 2*π*, *−π*/2 ≤ *θ*_2_ ≤ *π*/2).

The Average Linear Uncovered Length (ALUL), denoted by *ALUL*(*x*), introduced in [20,21], for the 2D space, gives an approximation of the average distance that can be made by a target, moving in 3D space, before being detected by the sensor network. The Average Linear Uncovered Length (ALUL) function can be defined by the following formula:

$$\mathrm{ALUL}\left(x\right)=\{\begin{array}{ll} 0, & \text{if}\;x\;\text{is covered}.\\ \frac{1}{{\left(2\pi \right)}^{2}}\int_{-\pi /2}^{\pi /2}\int_{0}^{2\pi }ℒ\left(x,\theta_{1},\theta_{2}\right)d\theta_{1}d\theta_{2}, & \text{otherwise}. \end{array}$$

More generally, when *A* is a subregion of the 3D domain under supervision, the Average Linear Uncovered Length related to *A*, *ALUL*(*A*), that a target can travel within *A* without been detected by a sensor is given by the expression:

(8) $$\mathrm{ALUL}\left(A\right)\equiv \frac{\int_{x\in A}\mathrm{ALUL}\left(x\right)\mathrm{dx}}{\left\|A\right\|},$$

where ||*A*|| is the volume of *A*.

the ALUL metric was developed to deal with a static deployment, which is not the case of our study. When a mobility model is implemented, the topology of the WSN is no longer static. To overcome this, we extend this notion so as to support sensor node mobility. The ALUL should also vary according to time and should use a function, denoted by the ℒ(*x*, *θ*, *t*), that defines the Linear Uncovered Length at location *x* with direction *θ*, at time *t*. Based on this reasoning, we define the metric *ALUL_m_*(*x, t*) representing the ALUL in a location *x* at time *t* and given by:

Due to sensor node mobility, the ALUL, over time, in a point *x* will be expressed by:

(9) $${\mathrm{ALUL}}_{m}\left(x\right)=\int_{0}^{\infty }{\mathrm{ALUL}}_{m}\left(x,t\right)\mathrm{dt}.$$

Finally, *ALUL_m_*(*A*) can be computed by Equation (8) by replacing *ALUL*(*x*) by *ALUL_m_*(*x*).

From the performance evaluation perspective, two important points should be highlighted:

- *ALUL_m_*(*A, t*) gives information about the coverage-preserving capabilities of the mobility model. It can be used to state whether the steady state is rapidly reached, and whether the mobility model affect the detection performance of the sensor network.
- *ALUL_m_*(*A*) provides information about the long-term behavior of the mobility model. It can be used to evaluate the impact of mobility on the possibility for a target to be undetected within the monitored region.

### 5.2. Mobility Models for Target Tracking

In this section, we show how the Voronoi cells can be used to implement target tracking using a sensor mobility model. In fact, we define two mobility models:

- The first model is called k-mobility model. Sensor nodes in this model move toward the regions where the hostile target is supposed to be and collaborate to keep the target controlled by k sensors all the time, To this end, the order k Voronoi diagrams are used and maintained all the time.
- The second model is called simplified model. It relies on estimating the uncovered zones within the Voronoi cells, using the ALUL metrics and moving sensor nodes toward the “uncovered zones”.

While the first model is triggered by the occurrence of targets, the second model aims at adapting the covered area so that the targets can be detected with higher probabilities. Obviously, the k-mobility model is more energy-consuming than the second since it encompasses the prediction of the target position and requires tracking using k sensor nodes. Therefore, we suppose that the second model can be used when energy resources become scarce. The performance of both models will be assessed in Section 7. Moreover, one can notice that the prediction function we are using is tightly related to the coverage of the zones where the targets are expected and that the mobility models assume that nearest sensor nodes can move to these zones while reducing the coverage of other zones where targets are not expected. In fact, the greater is the number of target detection signals, the better is the prediction precision to command sensor movements.

#### 5.2.1. The k-Mobility Model

In the following, we distinguish two cases: (a) a target crossing a *k−*covered area and (b) a target crossing non *k−*covered zone.

#### 5.2.2. For a Target Crossing a *k−*covered Zone

The mobility algorithm is triggered upon the detection of a target presence. Every detecting sensor sends its detection signal to the relevant intermediate sensor (called IS). The latter collects all detection signals, verifies their integrity, deduces the current zone where the target might be, estimates the positions of the target in the next of time slot, and commands k sensors to move to monitor the new zone to ensure tracking continuity.

Typically, the selected zone of target presence is taken among other zones (when more then k sensors detect the target presence). These zones are ordered according to the probability of presence of the target. The zone selected is the one presenting the highest probability among those which are *k−*covered.

The mobility algorithm is defined through five steps:

1. Assume that *k*′ sensors detect the target (*k*′ > *k*). The *k*′ sensors *s_i_*, 1 ≤ *i* ≤ *k*′, send their detection data *d_i_* to an intermediate node under the form:
  $$d_{i}=\left(r_{t,i},\theta_{t,i},\tau_{t,i},s_{i}\right)$$
  where *r_t,i_* = *δ*(*x_i_, z_t,i_*) is the Euclidean distance separating *s_i_* from the position *z_t,i_* of the target as seen by *s_i_, θ_t,i_* = (*α_t,i_*, *β_t,i_*) is the direction of the vector $\vec{z_{t,i}-x_{i}}$, and *τ_t,i_* is the detection instant.
2. In the case where detection signals are sent to different intermediate nodes; the intermediate coordinate to gather all signals (or at least *k* of them) at a unique node IS, which verifies first the authentication of the messages.
3. IS constructs:
  - The zone of target presence $Z_{t,i}^{\mathrm{tau}}$ for each sensor based the errors made for the values reported. This zone is delimited by the following eight points:
    $$(r_{t,i}\pm \Delta r,\left(\alpha_{t,i}\pm \Delta \alpha ,\beta_{t,i}\pm \Delta \beta \right)$$
    as defined by the estimated detection errors.
  - The most likely target presence zone *Z^τ^*(*t*). Several strategies can be used for this including selecting the largest intersection of k zones of the form $Z_{t,i}^{\mathrm{tau}}$. It can also be the largest union of k zones. Let *T* be the set of *k* sensors involved in the definition of *Z^τ^* (*t*).
  Then, IS computes the order k Voronoi cell *V^S^*(*T*). Obviously, it contains *Z^τ^* (*t*).
4. IS estimates the zone *Z*^*τ*,+^(*t*), where target *z_t_* is likely to be in the next time slot. Several strategies can be used for this estimation including extrapolation of older positions or some information related to target direction and speed. It also estimates the most likely new position of *z_t_*.
5. IS selects *k* sensors based on a specific criteria and order them to move towards *Z*^*τ*,+^(*t*) to increase its coverage. If no criteria is used, then the order goes to the sensors in *T*. A criteria can simply to reduce sensor movement.

When a criteria is applied for the selection of k sensors to cover the new position, some of the selected sensors (say *k*″ sensors) may belong to *T* and the other (say *k−k*″) have to be added among the neighbors of *T*. This situation is addressed in the following subsection.

#### 5.2.3. For a Target Crossing a Non *k−*covered Zone

In this case, only *k*′ (*k*′ ≤ *k*) detection signals are received by the intermediate sensor IS, which should proceeds at the construction of the probable current zone of presence of the target the way the preceding algorithms does. Then it starts the selection of the remaining (*k − k*′) required signals. Then, it orders the movement of the k sensor provide *k* monitoring to the target. For this purpose, IS executes the following steps:

1. IS computes the most likely zone of target presence let *z_t_* using the *k′* reports from *k′* sensors denoted by *s*_1_, ..., *s_k′_*.
2. For each *i* ≤ *k*′, IS selects the nearest *k* sensors to *s_i_*. It computes the related *k−*Voronoi cell $V_{i}^{\left(k\right)}$ and deduces the intersection $z_{t}\cap V_{i}^{\left(k\right)}$
3. For each *i* ≤ *k*′, IS gets the number of sensors *k_i_*”, 0 ≤ *k_i_*” *< k*, that have sent detection signals to IS.
4. IS classifies the *k−* Voronoi cells according to the value of *k_i_*”. The greater *k_i_*” is, the most important is the probability of presence of the target in $V_{i}^{\left(k\right)}$. A small value of *k_j_*” induces that the target is going in or out the cell $V_{i}^{\left(k\right)}$.
5. IS selects the nearest *k* sensors involved in $\partial V_{i}^{\left(k\right)}$, where *k_i_*” = *max*_*j*≤*k*′_*k_j_*, and guides the (*k −k*”) added sensors (among the nearest sensors to *s_i_*) to move towards $\partial V_{i}^{\left(k\right)}$. For that, it sends them a mobility instruction including the probability of presence of the target. A mobility instruction is defined by the 3-tuple.
  $$\left(r_{i},\alpha_{i},\pi_{i}\right)$$
  where *r_i_* ≥ (*s_i_, p*) such that $\forall q\in \partial V_{i}^{\left(k\right)}$, *δ*(*s_i_, p*) ≥ *δ*(*s_i_, q*). and $\alpha_{i}=\mathrm{argmax}\hat{xs_{i}y}$ where *x, y* ∈ *v_i_* and *v_i_* is the set of the vertices of the boundary ∂*V_i_*, *π_i_* = *k*”*/k* is the probability of presence of the target in $\delta V_{i}^{\left(k\right)}$.

To enhance coverage while keeping more mobility freedom, we implement a group mobility model in which ground sensors move in groups in order to preserve the *k−*coverage. To this purpose, for each mobility step, the sensors define randomly groups of *k* members for each, the latter are not required to be the nearest neighbors. Each group chooses randomly a head which chooses the first mobility step. The remaining members of the group take into account this choice to determine their next mobility step. By this way, every sensor’s mobility will depend on the integrating group. Furthermore, a sensor may move from one group to another in each mobility step. This model enables the definition of overlapping *k−*Voronoi groups which increases the guarantee of having a *k−*coverage.

#### 5.2.4. Simplified Mobility Model

We propose hereafter a mobility model which is based on the use of simple Voronoi diagram to identify and reduce coverage holes.

This model can serve to implement a mobility strategy where a sensor node looks for one or more neighbors that are at least 2*ρ*-distant from it. If such nodes exist, the sensor node moves toward the most distant neighbor, denoted by *n_f_*, with a distance $\frac{\delta \left(s_{i},n_{f}\right)-2\rho }{2}$.

The following result extends this strategy to the case where the monitored region is required to be *k*-covered using the simplified algorithm. It uses a set, denoted by *X*(*s_i_*, *V* (*S*)), which defined the set of intersection points expressed as follows:

(10) $$X\left(s_{i},V^{D}\left(S\right)\right)=𝔇\left(V^{D}\left(S\backslash \left\{s_{i}\right\}\right)\right)\cap \Gamma \left(s_{i},R_{s_{i}}\right),$$

where 𝔇, for a region *R* ⊆ **R**^3^, denotes the boundary of *R*.

For the sake of parsimony, we do not provide proofs for these corollaries in this paper.

**Lemma 5.1.** *For s_i_ in S, if |N*(*s_i_*, *V^D^*(*S*))*| < k, where |.| denotes set cardinality, then V^D^*(*s_i_*) *is not k-covered. For s_i_ in S, if |X*(*s_i_*, *V^D^*(*S*))*| < k, then V^D^*(*s_i_*) *is not k-covered.*

This lemma shows how simple Voronoi diagrams can be used to detect the coverage holes based on the distance between the sensor node and the edges of its Voronoi cell. It is based on the concept that the Voronoi tessellation is a partition of the points belonging to the monitored area according to their proximity to the sensor nodes. In other terms, if a point is not detected by the sensor node located at the generator of the Voronoi cell it belongs to, it cannot be detected by any other sensor node. If a sensor detects that the distance to one among the edges of its Voronoi edges is more than its coverage range, it has to move towards this edge to cover the corresponding hole. The uncovered can therefore be gradually reduced using this distributed strategy. However, a sensor node can detect that more than one of its Voronoi neighbors do not fulfill the condition of the lemma, it will therefore move towards the most distant neighbor.

The major advantage of this strategy, with respect to the advanced strategy, is that it relies on simple Voronoi diagrams to deal with *k*-coverage while the advanced model proposed in the previous subsection is based on order *k* Voronoi tessellations which are more complex to build.

A more accurate comparison between the two models will be carried out in the simulation section.

### 5.3. Multi-Target Tracking

The two tracking models presented in the above can be extended to the tracking of multiple targets. To describe the extension let us assume, for the sake of clarity, only two targets are detected by sensors in *S*. Let *z_t_* and *z_t_*_′_ be the reported positions.

The extension of the simplified model considers two cases:

- Only one node has detected the presence of the two targets: In that case, the sensor keeps monitoring one of the targets and invites the nearest neighbor to the second target to monitor the second and provides it with relevant information it collects.
- More than one node have detected the targets: In that case, two sensor among those that have detected the targets are selected to keep monitoring the targets independently.

On the other hand, the k-mobility model extends in following way: if d nodes detect the targets, these sensors are divided into two subsets, each in charge of monitoring one target, then the subsets are extended so that any of them contains k sensors.

## 6. Complexity Analysis of Coverage Management and Tracking

### 6.1. Complexity

In this section, we analyze the complexity of the different algorithms we have developed in the previous sections for the detect and locate holes or to repair coverage holes. Our approach to estimate the complexity can be based on the following metrics:

- The number of messages exchanged between the sensors during the execution of the algorithm.
- The number of additions and deletions of simplices to the Vietoris complex.
- The number of sensor movements made during the execution of an algorithm.

Some other operations can be added for a more accurate estimation of complexity. These metrics may include, for example, the number of storing operations made at the node level to update the related data structures. The messages exchanged during the execution of an algorithm can be of different types. In particular, they can be sent to a neighbor to tell it to change its status from internal (to the Vietoris complex) to external (*i.e.*, on the boundary of Vietoris complex). They also can be used to construct the initial boundary of Rips complex, or used to reduce the external boundary. They also can be sent after the retraction or the deflation of a simplex, or they are sent by a leader node the command a coordinated movement of sensors.

For the sake of clarity, we will focus on the complexity on the detection and counting of coverage hole. In this case, let *n* be the number of sensors in *S*, and *e* be the number of 1-simplices, *f* the number of 2-simplices, and *t* the number 3-simplices in RIPS complex of *S*). Let also *p* the number of vertices at the initial boundary of the Rips complex.

The number of messages sent during the execution of the algorithm should be lower or equal to the number of messages exchanged if all the polyhedra (external and internal) have been retracted first and that after deflation all the facets have been retracted. In that case, one can state that the number *N*_1_ of messages sent is given by:

N=p+(f−p)+(e−(f−p))=p+e≤|S|+e,

where *p* is the number of external vertices. This result can be deduced from the preceding and the fact that *p* ≤ *|S|*.

Now let us assume, without loss of generality, that the deployment of sensors (initial and current) guarantees that every node in the Rips complex of *S* has at most *v* neighbors (*v* is a fixed value coping with the volume of the area to monitor and the radius of coverage). Then, one can conclude that *e* is smaller than *v × |S|*, we deduce that

N≤n+v×|S|≤(v+1)n.

Let us notice that the assumption is not mandatory and a direct proof can be given. This shows that the algorithm to detect and count the coverage holes has a linear complexity.

### 6.2. Extending Results

The results presented in the previous sections can be extended in two dimensions: the type of the sensors and the occurrence of obstacles in the domain under monitoring.

**Coping with obstacles.** The algorithms developed in the preceding sections can be adapted to the occurrence of obstacles. Obstacles in monitored 3D areas may complicate seriously the role of monitoring sensors, increase their power consumption, and limit the coverage efficiency. Two particular objects have to be modified in our algorithms. First, the coverage holes that have to be counted should not contain obstacles. one can assume for this that the sensors are able to recognize an obstacle). Second, the mobility model used to increase coverage or to provide tracking should consider moving the sensor vertically as an alternative.

**Coping with semi spherical.** The algorithms developed in the preceding sections can be extended to semi-spherical sensors (sensor having a semi spherical covered area). It is worth noticing, at this point, that this type is sufficiently general to represent various sensors-base applications. In particular, the model can be used to represent fire and smoking-based sensors or camera-based sensors. To cope with semi-spherical sensors, one can notice that the concepts of Vietoris-rips and Voronoi diagram can be extended, so that coverage holes can be handles in a similar way. However, when repairing a hole, the mobility model of the sensor should include rotating a sensor to increase the coverage of a specific area by the sensor.

It is worth noticing that the additions made to the developed algorithms do not modify significantly the complexity of the algorithms. In particular, the complexity of the hole counter remains linear (as shown in the simulation discussed in the following section).

## 7. Experimental Results

In this section, we carry out a set of experiments to prove the efficiency of the proposed techniques. We first address the coverage hole problem by evaluating the performance of the higher-order Voronoi-based strategy for coverage optimization. To this purpose, we define a metric representing the ratio of uncovered area with respect to the total area of the monitored region. Second, We assess the target tracking approach by estimating the maximum linear distance that can be made by a hostile target without being detected. Finally, we evaluate the complexity of our coverage control and mobility techniques. We use the number of transmitted messages as a main criterion to estimate this complexity since data transmission consumes much more power than computational steps in WSNs.

### 7.1. Coverage Control and Hole Reduction

The first experiment aims at evaluating the hole reduction strategy based on three-dimensional Voronoi tessellations with spherical coverage. We define the following metric to evaluate the performance of hole reduction.

(11) $$\mu =\frac{\text{Sum\_of\_hole\_volumes}}{\text{Total\_volume\_of\_the\_monitored\_area}}.$$

**Figure 1.**
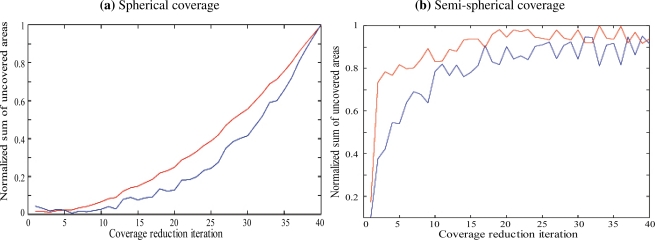
Evolution of the uncovered area proportion according to time.

Figure 1 shows the evolution of *μ* according to the number of iterations of the coverage hole reduction algorithm. We compared the Voronoi-based hole reduction strategy with the Homotopy-based strategy proposed in Section 4. It can be noticed that the increase in terms of normalized uncovered proportion is about 15% when the number of iteration is low. In addition, when the number of iteration exceeds 30, both approaches perform well since the normalized sum of uncovered areas becomes higher than 90%.

A similar experiment is conducted for vector guided sensors, assuming that at every step of the iteration, the mobility is provided along with an orientation of the vector to achieve better coverage. Figure 1 shows the evolution of *μ* according to the number of iterations of the coverage hole reduction algorithm and compares it to the Voronoi-based hole reduction strategy with the Homotopy-based strategy. One can conclude that while the homotopy-based approach is less complex, since linear for detection and localization, the Voronoi-based method reaches better results. In addition, a comparison between the results obtained for spherical sensors and semi spherical sensors shows the following:

- The approach performs better with spherical sensors for the first iterations. Indeed, the normalized uncovered proportion reaches 70%, with spherical sensors, after 10 iterations, while it stays under 10% for semi spherical sensors.
- The approach performs the same for both types of sensors after 30 iterations.

This can be explained by the fact that the density of sensors is the same for both types and, therefore, it takes more mobility for semi spherical sensors the coverage holes.

### 7.2. Mobility Modeling

The Average Linear Uncovered Length (ALUL), denoted by ℒ(*x*, *θ*), gives an approximation of the average distance that can be made by a target, moving in 3D space, before being detected by the sensor network. The metric *ALUL_m_*(*x, t*) representing the ALUL in a location *x* at time *t* is given by:

$${\mathrm{ALUL}}_{m}\left(x,t\right)=\{\begin{array}{ll} 0, & \text{if}\;x\;\text{is covered by a sensor}.\\ \frac{1}{{\left(2\pi \right)}^{2}}\int_{-\pi /2}^{\pi /2}\int_{0}^{2\pi }ℒ\left(x,\theta_{1},\theta_{2},t\right)d\theta_{1}d\theta_{2}, & \text{otherwise}. \end{array}$$

From the performance evaluation perspective, *ALUL_m_*(*A*) provides information on the coverage-preserving capabilities of the mobility model and the long-term behavior of the mobility model.

**Figure 2.**
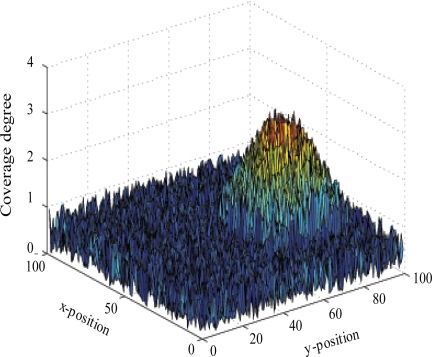
Local node density distribution after 5 mobility iterations.

In order to visually illustrate the performance of coverage reduction models, we use the local node density distribution that gives the number of sensors that cover every point of the monitored region. Figure 2 shows that, in the simple context where one target is moving within a 100 m^2^-size monitored zone, the coverage degree considerably varies according proximity to the mobile target. In fact, after 5 mobility steps, the local sensor density is less than 1 in regions that are far from the target location (which is (70,30)) and reaches 2.7 in points that are close to this target.

**Figure 3.**
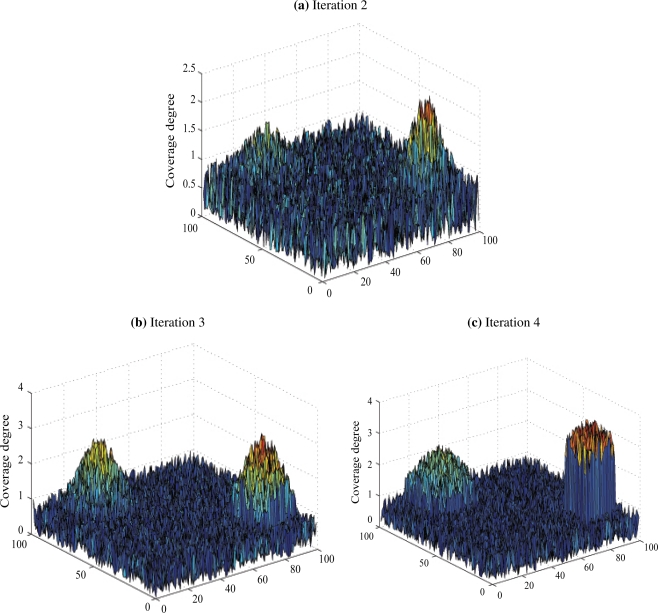
Illustration of 3 mobility iterations for a context where two targets are considered.

More interestingly, Figure 3 addresses the case where two targets are present within the region of interest. We notice that the sensors are initially uniformly distributed. The density then increases for the three following iterations in the regions where the targets are. In fact, this proves that our tracking scheme is precise enough to distinguish between the two different targets.

**Figure 4.**
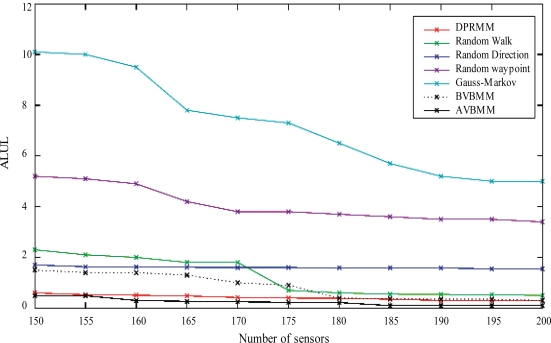
Evolution of the ALUL*_m_* metric according to time.

To confirm these results, we used the ALUL*_m_* metric to evaluate the evolution of the uncovered area with respect to time. In fact, this allows to know whether uncovered regions are created due to the density increase in the zones that are close to the target. We compared our scheme to four known mobility models, which are: the random walk model, the random waypoint model, the random direction model, and the Gauss-Markov model. The results of this comparison are depicted in Figure 4.

We notice that the proposed mobility models, denoted by Advanced Voronoi-based Mobility Model (AVBMM) and Distributed Voronoi-based Mobility Model (DVBMM), clearly outperform the existing models. They also return a better performance than the Density-Preserving Mobility Model. This is because the latter model, despite its ability to guarantee a nearly uniform node density within the monitored area, does not take into account the presence of hostile targets in the zone of interest.

### 7.3. Complexity Evaluation

In this subsection, we evaluate the communication overhead resulting from the proposed retract-based coverage control approach. To this end, we only consider the complexity of the detection and localization steps in our algorithm and do not address the complexity of the repair step, since the repair step complexity is mainly dependent on the first deployment. However, one can easily deduce that if the deployment guarantees that holes size do not exceed a threshold, then the linear complexity can be verified.

**Figure 5.**
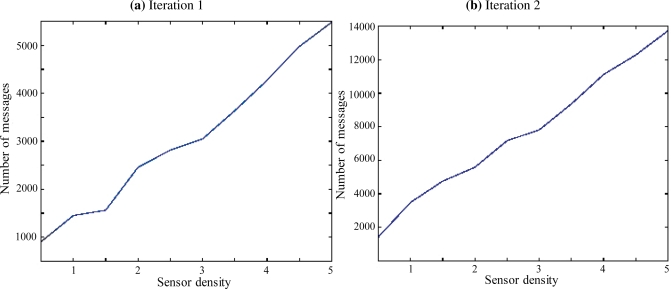
Illustration of complexity.

We considered that the dimensions of the monitored zone are 10 m × 10 m × 3 m. We varied the number of nodes deployed within this zone and we measured the number of messages required to setup our coverage control protocol. We first supposed that all sensor nodes have a spherical coverage of range 0.5 m. Figure 5 depicts the number of messages for densities ranging from 0.5 sensors per m^2^ to 5 sensors per *m*^2^. The major remark is that this number is nearly linear with respect to the number of sensors per area unit.

Moreover, we considered the case where sensors have semi-spherical coverage (with the same range). Figure 5 shows that the communication overhead is also linear in this situation but with a smoother slope. One can deduce the following statements from the aforementioned figures:

- The number of exchanged messages is independent of the density. It is close to 4 for the spherical sensors and 10 for semi spherical sensors. This fact may appear strange; however, one can notice that when a deployment is performed, the detection and location will only search for holes surrounded by the Vietoris space. The latter is reduced when the density is low.
- The number of messages exchanged by the semi spherical sensors for detection and localization is 2.5 times higher than the number observed for spherical. Two reasons can be mentioned for this. First, the area covered by a semi spherical sensors is half the area covered by spherical sensors. Second, the guiding vectors is randomly oriented.

## 8. Conclusion

This paper developed a low complexity approach to detect and localize sensing holes in 3D spaces. It also constructed efficient algorithms to repair holes and track (multiple targets). Our approach has built on two concepts, the Vietoris complex and the Voronoi diagram, and demonstrated that the technique called retraction by deformation achieves low complexity algorithms for the detection of coverage holes in WSNs.

Our approach can be easily extended to more general sensors, for which the Vietoris complex and the Voronoi diagram can be defined. Such sensors can be called conical sensors or vector guided sensors and can represent camera sensors.
